# Therapy with sirolimus in vascular anomalies: the experience of two Italian centers on 14 pediatric patients

**DOI:** 10.3389/fped.2024.1434493

**Published:** 2024-07-17

**Authors:** A. Neirotti, V. Barat, P. Coppo, R. La Selva, R. Manicone, R. Cotti, M. Sensini, A. Mussa, M. Gatto, F. Farri, M. E. Basso, F. Fagioli

**Affiliations:** ^1^ Pediatric Onco-Hematology, Stem Cell Transplantation and Cellular Therapy Division, Regina Margherita Children’s Hospital, Turin, Italy; ^2^ Pediatric Dermatology Unit, Regina Margherita Children’s Hospital, Turin, Italy; ^3^ Pediatric Radiology, Regina Margherita Children’s Hospital, Turin, Italy; ^4^ Pediatric Otorhinolaryngology, Department of Pediatrics, Regina Margherita Children’s Hospital, Turin, Italy; ^5^ Clinical Pediatric Genetics Unit, Department of Public Health and Pediatrics, University of Turin, Regina Margherita Children’s Hospital, Turin, Italy; ^6^ Division of Pediatrics, Department of Health Sciences, University of Eastern Piedmont, Novara, Italy; ^7^ ENT Division, University of Eastern Piedmont, Novara, Italy; ^8^ Division of Pediatrics, SS Annunziata Hospital, Savigliano, Italy

**Keywords:** sirolimus, vascular anomalies, safety, mTOR, *PIK3CA*

## Abstract

**Introduction:**

Vascular anomalies (VAs) constitute a heterogeneous group of tumors and malformations capable of inducing significant clinical events in specific patients, such as the compression of vital organs, pain, functional impairment, or acquired coagulopathy. Molecular investigations into the underlying mechanisms of VAs have unveiled the frequent involvement of the PI3 K/AKT/mTOR pathway. Sirolimus, a specific mTOR inhibitor, has emerged as a potential therapeutic agent; however, its routine clinical application in complex VAs is currently restricted by a lack of extensive clinical experience.

**Methods:**

Between 2015 and 2024, we administered sirolimus to 14 pediatric patients with various types of vascular anomalies in two Italian centers, subjecting them to clinical and instrumental follow-up to investigate its efficacy and the possible occurrence of adverse events.

**Results:**

An overall improvement in or stability of their vascular anomalies was reported by 86% of patients. We also assessed toxicity, noting a low prevalence of life-threatening adverse events: only one case of sepsis was reported in a patient with a severe clinical condition, and four cases of recurrent aphthosis (28%) were reported. The most common side effect was dyslipidemia, with 43% of patients developing hypercholesterolemia (21%) or hypertriglyceridemia (21%), although these patients generally did not reach severe levels.

**Discussion:**

In line with data in the literature, according to our experience, medical therapy with sirolimus should be considered in pediatric patients affected by vascular anomalies.

## 1 Introduction

Vascular anomalies (VAs) represent a spectrum of rare diseases classified into vascular tumors and malformations (1). This category includes vascular tumors characterized by endothelial cell proliferation and vascular malformations exhibiting mesenchymal and angiogenetic disorders. VAs are inherently congenital, persisting even when asymptomatic, and do not regress spontaneously. These malformations can be categorized based on a single vessel type, combined vascular components, or association with other non-vascular anomalies (2).

Clinical management of VAs requires a multidisciplinary approach involving different specialists for both diagnosis and therapy. Symptoms may vary depending on the type of VA and its location but generally include disfigurement, pain, infections, and coagulopathies involving both thrombotic and hemorrhagic aspects, potentially leading to organ dysfunction and, in severe cases, death (3, 4). In some cases of VAs, primarily kaposiform hemangioendothelioma, the Kasabach–Merritt phenomenon can occur, characterized by a life-threatening thrombocytopenia and consumptive coagulopathy (5).

Recent advancements in understanding VAs have emphasized the identification of common DNA variants, mostly somatic, implicated in their pathogenesis. Key pathways contributing to the genesis of vascular anomalies include the vascular endothelial growth factor (VEGF) pathway, the RAS/RAF/MEK/ERK pathway, the angiopoietin–TIE2 pathway, transforming growth factor-beta (TGF-beta) signaling, and the PI3K/AKT/mTOR pathway. These proteins play crucial roles in endothelial cellular growth, apoptosis, differentiation, proliferation, and the regulation of signaling and angiogenesis (4, 6, 7).

The recent understanding of the molecular basis of VAs suggested the increasing use of targeted therapies to inhibit overactive signaling pathways. Among the precision medicine approaches, sirolimus has been often used in treating VAs, and the medical literature, mostly focused on adult patients, has reported encouraging results so far, both in terms of clinical response and treatment tolerability.

The first studies reported an efficacy of oral sirolimus in treating VAs of nearly 80% (1, 8, 9) with a low rate of side effects. However, its employment as a topical medication remains controversial (3). Subsequent studies on sirolimus extended to pediatric patients with VAs reported comparable results, highlighting its positive effect, particularly on lymphatic and combined malformations (10–12).

Side effects reported in the literature are generally not severe and include mucositis in approximately 30% of patients, mild gastrointestinal symptoms, and dyslipidemia less frequently (10, 13). An increased susceptibility to infections is also described, although these infections are generally not severe nor life-threatening (14).

In general, the off-label use of sirolimus in children for VAs requires further investigation: the aim of this study is to contribute by providing further insight into this subject matter by reporting our experience and analyzing our case series of patients.

## 2 Materials and methods

We conducted a retrospective analysis of 14 pediatric patients who were under our care between 2015 and 2024 at the Pediatric Onco-Hematology Department of the Regina Margherita Children’s Hospital in Turin and the Division of Pediatrics of the Hospital Maggiore in Novara, Italy.

As sirolimus is employed off-label for treating VAs in childhood, its use was reserved for a few eligible patients. We included patients for whom a surgical approach proved impossible, ineffective, or difficult; those whose VAs highly impacted their quality of life due to pain or functional impairment; and those at risk of functional damage to an organ due to the growth of VAs. All included patients were between 0 and 20 years of age and able to take oral therapy. The exclusion criteria were refusal to participate in the study, pre-existing toxicity that prevented patients from taking sirolimus, inability or unreliability in taking oral therapy, the possibility of subjecting the patient to satisfactory surgery, and mild VAs without impairment of organ function or quality of life.

Eight patients (57%) were characterized at a molecular level by molecular analysis on bioptic VA samples to investigate mosaic and germline genetic variants in genes coding for proteins involved in PI3K/AKT/mTOR and RAS/MAPK/MEK pathways and genes of the vascular proliferation pathways. Three different custom next generation sequencing (NGS) panels were used including *PIK3R1*, *PIK3R2*, *PIK3CA*, *PTEN*, *PDK1*, *PDK2*, *KRAS*, *AKT1*, *AKT2*, *AKT3*, *RICTOR*, *MAPKAP1*, *MLST8*, *MTOR*, *IRS1*, *GAB1*, *GAB2*, *THEM4*, *MAPK8I1*, *PTPN11*, *RAPTOR*, *RASA1*, *TEK*, *TSC2*, *GNAQ*, *TSC1*, *DEPDC5*, *CCND2*, *NPRL3*, *GNA11*, *A2ML1*, *BRAF*, *CBL*, *HRAS*, *KRAS*, *MAP2K1*, *MAP2K2*, *NF1*, *NRAS*, *PTPN11*, *RAF1*, *RT1*, *SHOC2*, *SOS1*, *SPRED1*, *CCND2*, *GNA11*, and *RASA1* (15). Treatment approval from the Local Drug and Pharmacy Committee was obtained. This study received approval from the institutional review board (IRB) (No. 68301) on 17 June 2022 and was performed in line with the principles of the Declaration of Helsinki. Informed consent was obtained from all the individual participants included in the study and their parents.

The mean age at the start of treatment was 7.0 years ± 5.7 (range 0.7–19.3 years), and the average follow-up time was 37.9 months ± 22.9, with a range of 10–93 months.

Our patient group exhibited heterogeneity, encompassing

• five lymphatic malformations (three localized, two diffused),
• two venous malformations,
• four vascular tumors (two hemangioendotheliomas, one angioblastoma, one angiofibroma), and
• three combined VAs (one arteriovenous, two Klippel–Trénaunay syndrome cases).

Except for two patients who received sirolimus once a day, all the others received the therapy twice a day. In accordance with Società Italiana per lo Studio delle Anomalie Vascolari (SISAV) guidelines for VAs (16), we commenced sirolimus at a dosage of 0.8 mg/m^2^ per administration. The optimal sirolimus blood concentration varies in the literature, but the most frequently used concentrations in pediatric patients are 5–15 and 10–15 ng/ml (3, 12). Therefore, the dose was adjusted to achieve a target maintenance sirolimus blood concentration between 5 and 15 ng/ml. Treatment was also adjusted based on side effects and suspended in case of fever (body temperature >37.5°). All patients received prophylaxis with trimethoprim-sulfamethoxazole. Evaluation of the treatment response was based on both imaging techniques [Magnetic resonance imaging (MRI) and/or ultrasound (US)] and clinical findings, including patient-reported complaints related to pain, bleeding/oozing, and clinical assessment of VA swelling. The patients underwent systematic clinical and hematological follow-ups and imaging assessments (MRI and/or US) to evaluate the efficacy of the therapy and monitor related adverse events. Adverse events were categorized and graded using Common Terminology Criteria for Adverse Events Version 5.0 (CTCAE 5.0) (17). Data are available in online Mendeley repository https://data.mendeley.com/datasets/hsdwx9d6d4/1.

## 3 Results

The characteristics of all the patients are summarized in Table 1.

**Table 1:** Patients’ features and response to treatment.

** **	Age at start (y)	Sex	Location	Treatment before sirolimus	Duration of therapy (months)	Type of VA	Molecular investigation	Blood sirolimus target	Localization	Clinical improvement (symptoms/swelling)	Imaging modifications	SAEs	Treatment of SAEs
C1	12.5	M	Turin	Endoscopic exeresis	36	Vascular tumor	ND	In range	Maxillary sinus, nasopharynx	+	+	No	NA
C2	13.8	F	Turin	Surgery three times	34	Vascular tumor	Neg	Difficult to reach 5–15 ng/ml	Lower limb	+	=	Aphthosis (gII), herpetic keratosis (gII)	Local treatment with antiseptic and aciclovir
C3	2.8	M	Turin	Sclerotherapy two times	37	Lymphatic	ND	In range	Laterocervical	+	+	Hypertrig (gII), aphthosis (gII)	Omega-3 and local treatment with antiseptic
C4	1.7	M	Turin	Surgery	32	Vascular tumor	*PIK3CA*	In range	Lower limb	+	+	Hypertrig (gII)	Omega-3
C5	19.3	F	Turin	Surgery and sclerotherapy	16	Venous	ongoing	Difficult to reach 5–15 ng/ml	Cervicofacial	+	=/−	Aphtosis (gII), hyperchol (gII)	Local treatment with antiseptic and Omega-3
C6	6.5	F	Turin	Embolization	37	Venous	TEK/TIE2	In range, lower limit	Lower limb, gluteus	+	=	Hypertrig (gI)	Diet
C7	12.1	M	Turin	Surgery	12	Vascular tumor	ND	In range	lip	=	=	Hyperchol (gI)	Diet
C8	10.4	M	Turin	Surgery, sclerotherapy, and laser therapy	29	Combined in Klippel–Trénaunay	*PIK3CA*	In range	Limbs, facial	+	ND	Pimples (gI)	None
C9	1	F	Turin	Sclerotherapy	20	Lymphatic	ND	In range	Visceral diffused	−	−	No	NA
C10	5.1	M	Turin	Sclerotherapy and surgery	24	Lymphatic	Negative on pleural exudate	In range, fluctuating	Visceral diffused	−	−	Sepsis (gIV), hemorrhage (gIII), warts (gI), Bell’s palsy (gII)	Antibiotic and discontinuation of the drug
C11	3.1	F	Turin	Laser therapy	30	Combined in Klippel–Tréenaunay	*PIK3CA*	Difficult to reach 5–15 ng/ml	Lower limb	+	ND	Aphthosis (gII), hyperchol (gII), fatigue (gI), warts (gI), Bell’s palsy (gII)	Reduced dose until withdrawal
C12	3.1	M	Turin	None	9	Combined	TEK/TIE2	Just below lower limit	Back	+	+	No	NA
C13	0.7	F	Novara	Sclerotherapy two times	28	Lymphatic	ND	In range	Laterocervical	+	+	No	NA
C14	5.6	M	Novara	Sclerotherapy	4	Lymphatic	ND	Just below lower limit	Laterocervical	+	+	No	NA

SAE, side adverse event; M, male; F, female; Hypertrig, hypertriglyceridemia; hypercol, hypercholesterolemia; g, grade; +, improvement; =, stability; −, worsening; NA, not applicable; ND, no data; Neg, negative.

The mean duration of sirolimus therapy was 24.8 months ±10.5 (range 4–37 months), and currently, 7 out of the 14 patients are still undergoing therapy (50%). Among these, in three patients (21%), it was difficult to maintain appropriate blood levels of sirolimus due to the onset of side effects for which we could not increase the dosage, while three patients (21%) showed values fluctuating around the lower limit. At the end of the observation period, 12 out of 14 patients (86%) are alive, while 2 (14%) have deceased.

In 13 patients (93%), we attempted an ineffective invasive approach or local treatment before initiating medical therapy.

Currently, seven patients (50%; cases 1, 2, 3, 5, 6, 12, and 14) are under treatment. Two patients (14%; cases 9 and 10) discontinued the therapy due to inefficacy, two patients (14%; cases 4 and 8) discontinued the therapy to start another tailored drug following molecular tests results, one patient (7%; case 7) decided to withdraw his informed consent, one patient (7%; case 13) interrupted treatment for medical reasons (stable lesion), and one (7%; case 11) discontinued because of multiple concomitant adverse events, probably related to the treatment.

Out of 14 patients, 8 (57%) underwent molecular investigations, with positive results in 5 cases. Pathogenic variants were found in *TEK* gene (p.Leu914Phe in case 6, p.Thr1106Glufs*6 in case 12) coding for protein TIE2 and in *PIK3CA* gene (p.Arg108His in case 4, p.Gln546Lys in case 8, and p.Glu542Lys in case 11) coding for PI3K.

### 3.1 Efficacy

As shown above, 12 patients (86%) reported an overall improvement or stability of their VAs, with 11 cases (78%; cases 1–6, 8, 11–14) showing improvement in symptoms, swelling, and/or imaging findings, while one case (7%; case 7) remaining stable. On the other hand, sirolimus failed to induce an improvement in two patients (14%; cases 9 and 10), both with a severe syndromic condition and visceral involvement, resulting in death due to disease progression.

Of the 12 patients responding to treatment, effectiveness was proved by imaging in six cases (43%; by MRI in cases 1, 3, 4, and 13 and by US in cases 12, 13, and 14), obtaining a reduction in the size of VAs. Case 11 reported a worsening after discontinuation of sirolimus, despite an improvement in the clinical picture during therapy.

Treatment efficacy in reducing symptoms was documented in 11 cases (78%): cases 2, 6, and 8 reported reduced pain; case 1 experienced a disappearance of headaches; and cases 3, 4, 5, 11, 13, and 14 exhibited an improvement in swelling or cutaneous involvement. Case 11 reported fewer bleeding episodes, and case 12 noted the end of thrombotic episodes.

Upon stratifying our cases, all patients (100%) with combined or venous vascular anomalies demonstrated improvement under treatment, whereas patients with vascular tumors showed improvement in three out of four (75%) and substantial stability in one case (case 7). All three cases (100%) of localized lymphatic malformations responded to treatment, while the only notable therapeutic failure occurred in two patients with diffuse lymphatic malformations with visceral involvement, where organ functionality was seriously impaired before initiating sirolimus therapy.

Of the three patients in whom we encountered difficulties in maintaining sirolimus within the target blood range (21%; cases 2, 5, and 11), one discontinued the drug intake due to adverse events (case 11), while the other two continued as drug withdrawal resulted in a rapid worsening of symptoms.

All patients (100%) with a mutation for genes coding for proteins involved in the PI3K/AKT/mTOR pathway reported a clinical response after initiation of sirolimus therapy.

### 3.2 Safety

As concerns safety, we reported only one major infectious event (sepsis with pelvic hemorrhage) in patient 10; this was an isolated episode occurring in a child with a very complex clinical picture. Dyslipidemia was the most common adverse event (46%), with three cases (21%) of hypertriglyceridemia (two of grade 2 and one of grade 1) and three cases (21%) of hypercholesterolemia (two of grade 2 and one grade 1). The second most common side effect was recurrent aphthosis (four patients; 28%), but it was not severe in all cases. In addition, we observed two cases (14%) of reversible Bell's palsy and two patients (14%) with a recurrence of warts. In our cohort, we treated aphthosis with local antiseptic therapy, grade 1 dyslipidemia with diet, and grade 2 dyslipidemia with omega-3. During sepsis in patient 10, we suspended administration of sirolimus, while in patient 11, who showed multiple side effects simultaneously, we progressively reduced the dose administered until discontinuation.

## 4 Discussion

### 4.1 Efficacy

We report here a case series of children with VAs treated with sirolimus. Our results are encouraging and consistent with those of earlier scientific studies on 57 and 41 patients of all ages (1, 9), reporting success rates of 85% and 80%, respectively. The results also align with the findings of Ji et al. (10), who observed a 77.8% response to sirolimus in 126 pediatric patients after 12 months, and with the results of Zhou et al. (18), who reported a 92.2% response to sirolimus in 154 patients with kaposiform hemangioendothelioma.

Compared to previous data in the literature, we observed differences in response rates among the different subtypes of VAs: improvement or stability was observed in all patients with venous, lymphatic (localized), combined malformations and vascular tumors, while previous studies found differences in response rates among the various subgroups of VAs. In fact, the study by Ji et al. (10) reported responses mainly in lymphatic, venous, venolymphatic malformations and kaposiform hemangioendothelioma, while Maruani et al. (11) reported responses in terms of both symptom regression and volume reduction in lymphatic malformations, only in symptoms in cases of combined malformations, and poor responses in venous malformations.

The response to sirolimus in our case series was poor only in cases with visceral lymphatic malformations, but it includes two patients with very severe organ function impairment at the initiation of therapy. Except for these two patients in whom we did not obtain satisfactory results, the others showed positive outcomes regardless of the type of VA. In addition, we observed that, regardless of the type of VA, the best results in terms of symptom reduction and imaging were achieved in patients who started sirolimus at a younger age. This is likely due to the arrest of the anomaly's growth during the patient's growth phase, where the malformation remains stable as the patient continues to grow.

### 4.2 Safety

Our data concerning the side effects of sirolimus reflect those in the literature, although we evidenced a slightly lower incidence of oral mucositis than the expected rate. The review by Zhang et al. (13), which includes nine studies involving 575 pediatric patients receiving sirolimus, indicated a 20.52% occurrence of mucositis considering all the therapeutic indications, with a prevalence of 33.9% in patients with VAs, while the study by Ji et al. (10) reported a 37.0% occurrence. Conversely, we observed a higher rate of patients developing dyslipidemia (43%) compared to that has been reported in the literature. Zhang et al. (13), in fact, reported dyslipidemia in only 6.26% of cases. Only in one situation did adverse events lead us to stop the treatment, primarily due to the concomitance of side events rather than their severity (case 11).

In our patient group, we did not report infections due to *Pneumocystis carinii*, but it is to note that all patients took trimethoprim-sulfamethoxazole, as this infection has been described during sirolimus treatment, especially without prophylaxis (19).

Finally, it is also important to note that we found a high rate of patients positive for specific variants within the several cellular pathways investigated; such cases might deserve targeted treatment, and therefore, we recommend that patients with VAs must undergo genetic testing for somatic variants as soon as possible.

### 4.3 Molecular findings

In our sample, as described above, we performed molecular investigations and found five pathogenic variants (two in *TEK/TIE2* and three in *PIK3CA* gene). Literature data suggest somatic mutations in the PI3K/AKT/mTOR pathway primarily in patients with lymphatic and venous VAs and in those with syndromic conditions (Klippel–Trénaunay syndrome, congenital lipomatous overgrowth, vascular malformations, epidermal nevi and scoliosis/skeletal/spinal anomalies (CLOVES) syndrome, blue rubber bleb nevus syndrome, etc.) (4). The patients we described, in whom we found mutations in genes involved in the PI3K/AKT/mTOR pathway, reflect the literature in three cases (two of Klippel–Trénaunay syndrome and one of venous malformation), while one case of arteriovenous malformation and one case of kaposiform hemangioendothelioma showed mutations in *TEK* and *PIK3CA* genes, respectively. However, arteriovenous malformations and kaposiform hemangioendothelioma are described as being mainly associated with somatic mutations in the RAS/MAPK/MEK pathway (4).

### 4.4 Conclusions

Considering the good response rate with the restrained occurrence of tolerable toxicity, we can cautiously assert that sirolimus therapy should be considered in pediatric patients with VAs. Nevertheless, while side events are relatively few, they are not negligible. Clinicians should carefully consider the risk–benefit ratio at the initiation of therapy, determining which cases of VAs may benefit from sirolimus.

This series has obvious limitations, mostly regarding the retrospective nature of the study and the limited number of observations. Although our data, in conjunction with previous literature, support the use of sirolimus for VAs, further studies with larger samples and higher quality are necessary to enhance our understanding of this drug.

## Data Availability

The datasets presented in this study can be found in online repositories. The names of the repository/repositories and accession number(s) can be found in the article/Supplementary Material.
